# Correspondence

**Published:** 1907-01

**Authors:** Herbert E. Smith

**Affiliations:** Dean of the Faculty of Medicine. Yale University, Medical Department, New Haven


### CORRESPONDENCE.

---

**Professor Herbert E. Smith, Dean of the Faculty of Medicine of Yale University, States the Requirements for Admission to the Yale Medical School, beginning September, 1909. He thinks Two Years of Collegiate Work an Adequate Preparation for Professional Study.**

Editor Buffalo Medical Journal.

Sir: Believing that you are interested in every effort to improve the grade of students entering our medical schools, I am sending you a statement of the requirements for admission to the Yale Medical School which have just been announced by the corporation of Yale University as applying to the ninety-seventh annual session beginning in September, 1909.

The essential points of the requirements are that the minimum entrance qualification will be two years' work of college grade, and that the preparation must include inorganic chemistry, physics and general biology. In view of the fact that a student in Yale College may by a proper choice of subjects secure both the B.A. and M.D. in six years, the effect of these regulations will be doubtless that the great majority of those receiving the degree of M.D. at this University will receive it as a second degree. We expect this to be the case, and we hope that many will pursue more than the minimum of two years of college work. We believe, however, that the preparation involved in the college entrance requirements, and two years of collegiate work is an adequate preparation for professional study, and we do not think it desirable to make it impossible for a student to proceed to the degree with this minimum requirement in case, for any reason, he elects to do so.

The requirements as they will apply to college graduates, and to non-graduates are as follows:

I. Candidates who have received degrees in arts or science from approved universities or colleges, will be admitted on presenting their diplomas or other satisfactory testimonials.

II. Other candidates must present evidence that they have complied with the entrance requirements of some collegiate institution of good standing, or have passed equivalent examinations before some recognized examination board such as the College Entrance Examination Board. They must also present evidence that they have performed, with credit, the equivalent of at least two full years of work of collegiate grade of fifteen hours per week. Such evidence may be furnished by certificate from an institution of good standing. Candidates who have not attended institutions able to give this certificate but who have otherwise fitted themselves for the study of medicine by work of corresponding grade, may qualify by examination in this University on payment of a fee of $10.00.

All candidates for admission must furnish evidence that they have a satisfactory preparation in physics, general inorganic chemistry, and general biology.

HERBERT E. SMITH,
Dean of the Faculty of Medicine.

YALE UNIVERSITY, MEDICAL DEPARTMENT,
NEW HAVEN, DECEMBER 1, 1906.

---

PRURITUS ANI.—T. Chittenden Hill says that by far the most important causes of pruritus ani are superficial ulcerations or abrasions of the anal canal. This lesion is very constant and should always be looked for. The writer then discusses various other conditions associated frequently with this affection, among which are catarrhal diseases, hemorrhoids, and polyps. Appropriate treatment must be applied to the unnatural skin before much amelioration of the itching can be expected. After defecation the patient should cleanse thoroughly the anal region, preferably with absorbent cotton which has been wrung dry of some antiseptic solution. An anal pad held in place by a T bandage will protect the parts from friction and irritation of the discharge. Nitrate of silver and citrine ointment are excellent applications in most cases. For delicate skins the following ointment may be substituted for the citrine: red oxide of mercury three drachms, Venice turpentine one ounce, lanolin three ounces.—*Medical Record,* December 22, 1906.

